# Editorial: Neuroergonomics in Human-Robot Interaction

**DOI:** 10.3389/fnbot.2022.1006103

**Published:** 2022-09-06

**Authors:** Giacinto Barresi, Chang S. Nam, Ehsan T. Esfahani, Michela Balconi

**Affiliations:** ^1^Rehab Technologies, Istituto Italiano di Tecnologia, Genoa, Italy; ^2^Edward P. Fitts Department of Industrial & Systems Engineering, North Carolina State University, Raleigh, NC, United States; ^3^Human in the Loop Systems Laboratory, Department of Mechanical and Aerospace Engineering, University at Buffalo, Buffalo, NY, United States; ^4^International Research Center for Cognitive Applied Neuroscience (IrcCAN), Catholic University of the Sacred Heart, Milan, Italy; ^5^Research Unit in Affective and Social Neuroscience, Department of Psychology, Catholic University of the Sacred Heart, Milan, Italy

**Keywords:** neuroergonomics, Human-Robot Interaction, human factors, robotics, human-centered technology

Neuroergonomics (Parasuraman, 2003; Ayaz and Dehais, 2021; Gramann et al., 2021) can be quite impactful to investigate and improve Human-Robot Interactions (HRIs) (Scotto di Luzio et al., 2018; Roy et al., 2020; Rosén, 2021), analyzing and affecting the neural processes of any individual interacting with a smart machine that can work as collaborator, tool, or even extension of its user in ecologically valid contexts. Accordingly, we can achieve a “neuroergonomic robot”: usable, acceptable, safe, and minimally demanding in terms of mental workload according to indices of neural activity, considered as the antecedents of any experience and behavior. A robotic system may exploit these indices to recognize the individual conditions for adjusting its activity to ameliorate the human-machine system performance alongside the safety and the wellbeing of the user. The collection of papers presented in this Research Topic propose examples of investigations and concepts on neuroergonomics in HRI, suggesting further breakthroughs in user-centered robotics.

For instance, a manuscript introduces relevant topics in neuroergonomics that highlight how the roots of this discipline also reach the ground between the discoveries in neuroscience and the innovations in neuroengineering. *Direct Communication Between Brains: A Systematic PRISMA Review of Brain-To-Brain Interface*, Nam et al. discussed the current state of brain-to-brain interface (B2BI) technologies and its potential in transmitting information between two individuals through a brain-computer interface (BCI) and a computer-brain interface (CBI). Such a revolutionary concept can lead to novel neuroergonomic paradigms of collaboration across robotic devices and multiple users. This review definitely remarks the importance of the neurocognitive and neurobiological concepts in this field, as presented about framework for teaching and training in *The Complexity of Remote Learning: A Neuroergonomical Discussion* by Cassioli and Balconi. However, pondering the individual and contextual requirements in this path also needs techniques of other branches of human factors. Accordingly, Bevilacqua et al. presented their *Design and development of a scale for evaluating the acceptance of social robotics for older people: The Robot-Era Inventory*: in this manuscript, the authors introduce a set of scales for assessing social assistive robots cooperating with older adults. This inventory can certainly work in synergy with psychophysiological measures of elderly reactions during the interaction with a device.

This would be especially advantageous in the domain of social HRI, which encompasses neuroscientific studies like the one of Marchesi et al.: *I Am Looking for Your Mind: Pupil Dilation Predicts Individual Differences in Sensitivity to Hints of Human-Likeness in Robot Behavior*. Through an experimental investigation involving the humanoid robot iCub, the authors demonstrate how patterns of pupil dilation and response time can unveil individual biases in interpreting the behavior of a human-like artifact, perceived as an intentional agent. These results may lead to innovations in the design of socially attuned humanoids. This neuroscientific approach could surely be extended through the adoption of portable neurotechnologies, as argumented by Cassioli et al. in *Human–Co-Bot Interaction and Neuroergonomics: Co-Botic vs. Robotic Systems*. The approach proposed by these authors is especially peculiar for demonstrating the advantages of organizational neuroergonomics on collaborative robotics. Such a perspective remarks how neuroergonomics in HRI can express its own contribution across multiple branches of human factors. Another example of this versatility, considering both physical and cognitive ergonomics, is constituted by a study authored by D'Antonio et al. and titled *Robotic Assessment of Wrist Proprioception During Kinaesthetic Perturbations: A Neuroergonomic Approach*. In this research, the authors present a refined methodology, based on a haptic neuroergonomic wrist device, for investigating the effects of systematic perturbations on the user's proprioceptive and kinaesthetic acuity. Their results are particularly valuable for the clinical evaluation of neurological damages: such a delicate field requires levels of performance, reliability, and robustness of robotic devices that just the approaches of human factors—including neuroergonomics— can guarantee. This study also dedicate special attention to its methodological appropriateness, a critical point in any interdisciplinary domain.

Indeed, we surely need to design and implement novel solutions for research, as discussed by Savković et al. in *Development of Modular and Adaptive Laboratory Set-Up for Neuroergonomic and Human-Robot Interaction Research*. The authors describe their specialized infrastructure for assessing workers' performance, safety, wellbeing, and experience, considering anatomical, anthropometric, physiological, and biomechanical data. However, devising innovative equipment also requires to explore groundbreaking concepts to introduce novel methodologies. For instance, Del Vecchio et al. wrote *Peripheral Neuroergonomics – An Elegant Way to Improve Human-Robot Interaction?* to remark how most non-invasive human-robot interfaces based on the peripheral nervous system seems to offer an appropriate interpretability. This makes them currently advantageous over solutions (especially the invasive ones) collecting data from the central nervous system. Fostering synergistic approaches based on peripheral neural signals alongside central ones and motor data seems particularly promising, and it can become imperative for the twinning strategy presented by Barresi et al. in *Beyond Digital Twins: Phygital Twins for Neuroergonomics in Human-Robot Interaction*. This paper proposes a concept to replicate a remote human-robot system through a partially virtual and partially mechatronic solution, exploiting “phygital” features that make it more reliable and easy to be manipulated by a person assessing its potential states. Such a twinning design enables “metalaboratories” for investigating the conditions of the remote robot users in their context according to multimodal data collected by wearable sensors. The need of heterogenous information is furtherly highlighted by Corti in *The Role of Neuroergonomics in the Design of Personalized Prosthesis: Deepening the Centrality of Human Being*. The author points at the value of a quanto-qualitative approach to bridge phenomenological and the neuroscientific concepts and methods to investigate relevant topics within the domain of neuroergonomics in HRI like the prosthetic embodiment. This is a compulsory step for understanding a multifaceted system based on the interactions between humans and their robotic collaborators, tools, and extensions.

Overall, this Research Topic offered the opportunity to collect insightful contributions from experts in different domains (from psychology to engineering, from neuroscience to philosophy), foreseeing neuroergonomic (even neurosensitive) robots as a step-change in human-centered technology transfer within a greater journey for achieving practicality and sustainability in HRI.

## Author contributions

All authors contributed to the manuscript, revised, and approved its final version.

## Conflict of interest

The authors declare that the research was conducted in the absence of any commercial or financial relationships that could be construed as a potential conflict of interest.

## Publisher's note

All claims expressed in this article are solely those of the authors and do not necessarily represent those of their affiliated organizations, or those of the publisher, the editors and the reviewers. Any product that may be evaluated in this article, or claim that may be made by its manufacturer, is not guaranteed or endorsed by the publisher.
